# Application of IL-36 receptor antagonist weakens CCL20 expression and impairs recovery in the late phase of murine acetaminophen-induced liver injury

**DOI:** 10.1038/srep08521

**Published:** 2015-02-17

**Authors:** Patrick Scheiermann, Malte Bachmann, Lorena Härdle, Thomas Pleli, Albrecht Piiper, Bernhard Zwissler, Josef Pfeilschifter, Heiko Mühl

**Affiliations:** 1 pharmazentrum frankfurt/ZAFES, University Hospital Goethe-University Frankfurt; 2 Clinic for Anesthesiology, University Hospital Ludwig-Maximilians-University Munich; 3 Medical Clinic I, University Hospital Goethe-University Frankfurt, Germany

## Abstract

Overdosing of the analgesic acetaminophen (APAP, paracetamol) is a major cause of acute liver injury. Whereas toxicity is initiated by hepatocyte necrosis, course of disease is regulated by mechanisms of innate immunity having the potential to serve in complex manner pathogenic or pro-regenerative functions. Interleukin (IL)-36γ has been identified as novel IL-1-like cytokine produced by and targeting epithelial (-like) tissues. Herein, we investigated IL-36γ in acute liver disease focusing on murine APAP-induced hepatotoxicity. Enhanced expression of hepatic IL-36γ and its prime downstream chemokine target CCL20 was detected upon liver injury. CCL20 expression coincided with the later regeneration phase of intoxication. Primary murine hepatocytes and human Huh7 hepatocellular carcinoma cells indeed displayed enhanced IL-36γ expression when exposed to inflammatory cytokines. Administration of IL-36 receptor antagonist (IL-36Ra) decreased hepatic CCL20 in APAP-treated mice. Unexpectedly, IL-36Ra likewise increased late phase hepatic injury as detected by augmented serum alanine aminotransferase activity and histological necrosis which suggests disturbed tissue recovery upon IL-36 blockage. Finally, we demonstrate induction of IL-36γ in inflamed livers of endotoxemic mice. Observations presented introduce IL-36γ as novel parameter in acute liver injury which may contribute to the decision between unleashed tissue damage and initiation of liver regeneration during late APAP toxicity.

Tissue injury associated with inappropriate application of the analgesic acetaminophen (APAP, paracetamol) is a leading cause of acute liver failure and thus considered a major burden for health care systems worldwide^1^. From a conceptual point of view, experimental APAP-induced liver injury is a valid model for studying acute diseases initiated by parenchymal cell death, but likewise regulated by innate immunoactivation in response to release of alarmins from necrotic or necroptotic cells^2^,^3^,^4^. Alarmins that supposedly contribute to APAP-induced liver injury include DNA^5^,^6^ and ATP^7^ as well as protein factors such as high-mobility group box protein-1 (HMGB1)^8^,^9^, and histones^10^. With the exception of ATP activating P2X_7_ receptors^7^, aforementioned alarmins may aggravate disease by action on toll-like receptors (TLRs)^2^,^3^,^11^. Independent reports relate severity of APAP-induced liver injury to activation of the innate TLR system mediating detrimental inflammation. This specifically applies to TLR4^10^,^12^,^13^, TLR3^14^, and TLR9^5^,^9^. However, the role of the innate immune system and related cytokine production in APAP-induced liver damage is more complex and definitely includes the potential to initiate protective mechanisms that associate with repair and regeneration^15^. Indeed, if tissue damage stays below a threshold, resolution prevails and prevents loss of organ function by initiating compensatory proliferation, repair, and regeneration. Of note, crucial protective/pro-regenerative functions of liver macrophage/dendritic cell/Kupffer cell populations are evident in the context of murine APAP intoxication^16^,^17^. This complexity is also reflected by partly disparate results obtained in different studies evaluating fine-tuning of APAP toxicity. For example, while confirming previous data on the pathogenic action of HMGB1^8^ and TLR9^5^, a recent report did not support a role for TLR4 in mediating APAP-induced tissue damage^9^.

Some members of the interleukin (IL)-1 cytokine family^18^, for example IL-1α and IL-1β, are supposed to be upregulated by alarmins during APAP-induced liver injury. However, the role of IL-1 in APAP toxicity is actually not uniformly assessed. Either pathogenic action^5^,^19^, no significant function^20^, or protection^21^ by IL-1 has been observed in APAP-induced liver injury. A further cytokine of the IL-1 family able to efficiently activate epithelial (-like) cells is IL-36γ^22^. This cytokine, formerly known as IL-1F9^23^, shows characteristic properties shared by several IL-1 family members such as absence of a conventional signal peptide^18^, the necessity for proteolytic maturation to acquire full biological activity^24^, and usage of the IL-1 receptor accessory protein (IL-1RAcP) as one component of its heterodimeric receptor^18^,^25^. The other receptor component, IL-1 receptor related protein-2 (IL-1Rrp2), specifically binds to IL-36γ and its siblings IL-36α or IL-36β and initiates, in cooperation with IL-1RAcP, signaling via nuclear factor (NF)-κB and mitogen-activated protein kinases^25^. Besides mononuclear phagocytes and T cells^26^,^27^, especially cells of epithelial origin are sources and targets of IL-36γ^22^,^28^,^29^. Accordingly, IL-36γ has been linked to the pathogenesis of psoriasis^22^. Biological activity of IL-36 cytokines is controlled by IL-36 receptor antagonist (IL-36Ra) which tightly binds IL-1Rrp2 but lacks the potential to recruit IL-1RAcP. Excess of IL-36Ra thus blocks IL-36 function in a manner similar to the action of IL-1Ra on IL-1 biological activity^24^. Furthermore, IL-38 is capable of inhibiting IL-36γ by analogous action^30^. The relevance of IL-36 in pathophysiology is underscored by aggravated experimental psoriasis upon IL-36Ra deficiency^31^,^32^. Even more impressive is the association between non-functional IL-36Ra and the development of clinical generalized pustular psoriasis, a most severe entity of dermal inflammation^33^.

Since specific information on that matter is currently lacking, we set out to investigate the significance of IL-36 cytokines in the hepatic compartment with focus on APAP-induced liver injury.

## Results

### Expression of IL-36γ in murine APAP-induced liver injury and inflamed hepatocytes

**Figure 1:**
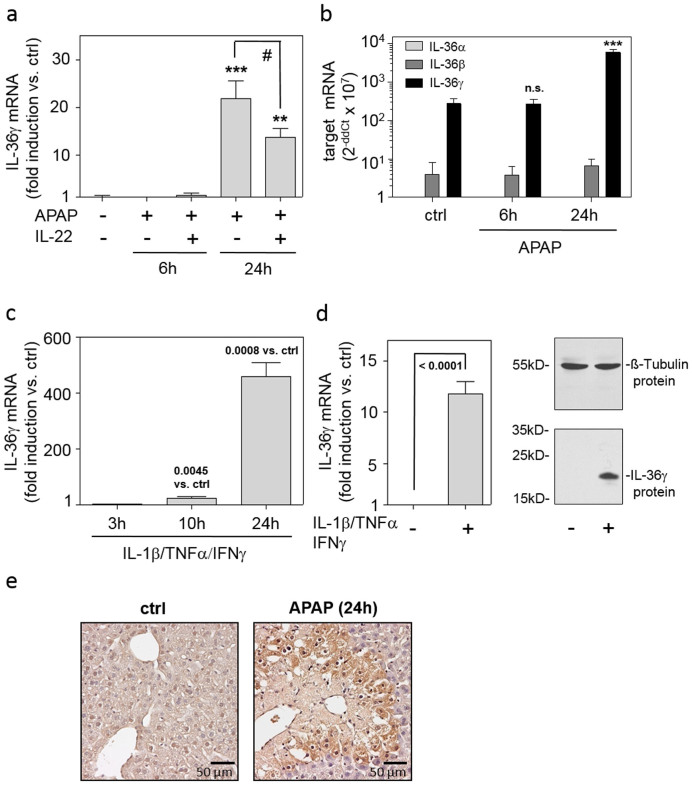
Expression of IL-36γ in murine APAP-induced liver injury and inflamed hepatocytes. (ab) Mice received PBS (n = 6) or APAP or (where indicated) APAP/IL-22 (6 h (n = 6), 24 h (n = 9)). (a) Hepatic IL-36γ mRNA was determined by realtime PCR. Target mRNA was normalized to that of GAPDH (means ± SEM *versus* ctrl; **p < 0.01, ***p < 0.001 *versus* ctrl; #*p* < 0.05). (b) Hepatic IL-36αβγ mRNAs were determined by realtime PCR. Target mRNA was normalized to that of GAPDH and is shown as raw data (2^−ddCt^ × 10^7^; means ± SEM; ***p < 0.001 *versus* ctrl of the same target gene; n.s., not significant). (ab) Statistical analysis, one-way analysis of variance with post hoc Bonferroni correction. (c) Murine primary hepatocytes were kept as unstimulated control (ctrl) or stimulated with IL-1β/TNFα/IFNγ (each 50 ng/ml). IL-36γ mRNA expression was determined by realtime PCR. Target mRNA was normalized to that of GAPDH (means ± SEM *versus* ctrl; n = 3). (d) Huh7 cells were kept as unstimulated ctrl or stimulated with IL-1β/TNFα/IFNγ (each 50 ng/ml). After 3 h (left panel) or 24 h (right panel), IL-36γ mRNA (left panel) or protein (right panel) was determined by realtime PCR or immunoblotting, respectively. Target mRNA was normalized to that of GAPDH (means ± SD *versus* ctrl; n = 8). One representative immunoblot of five independently performed experiments is shown. (cd) Statistical analysis, Student's t-test *versus* ctrl at the respective time point. (e) Representative murine liver immunohistochemistry of IL-1Rrp2 at 24 h after APAP application (left panel: ctrl; right panel: APAP).

Since IL-36γ is a novel parameter potentially determining the biology of ‘epithelial-like' hepatocytes and thus APAP-induced liver injury, we set out to investigate its expression in this setting. As shown in Fig. 1A, upregulation of hepatic IL-36γ expression was detectable at 24 h, but not at 6 h, after application of APAP. Notably, amelioration of APAP-induced liver disease by administration of recombinant IL-22^34^,^35^ associated with modulation of hepatic IL-36γ (Fig. 1a), suggesting that expression of IL-36γ may link to disease severity. To assess the specificity of IL-36 expression in APAP-induced liver injury, hepatic IL-36α and IL-36β were likewise determined. Notably, IL-36γ was by far the most abundantly expressed IL-36 cytokine in this model of acute liver damage (Fig. 1b). We previously reported on IL-36γ expression in primary human monocyte-derived dendritic cells or M1 macrophages under the influence of IL-1β/TNFα/IFNγ^26^. Yet, a characteristic feature of IL-36γ biology is its expression in activated cells of epithelial origin such as keratinocytes^28^ and lung epithelial cells^29^. Therefore, expression of IL-36γ was investigated in primary murine hepatocytes and human Huh7 hepatocellular carcinoma cells. Here, we demonstrate upregulation of IL-36γ by inflammatory IL-1β/TNFα/IFNγ in both cell types (Fig. 1cd). Notably, expression of IL-1^5^,^20^ as well as TNFα^14^,^34^,^36^,^37^,^38^ and IFNγ^37^,^38^,^39^ has been detected in liver tissue undergoing APAP-induced injury. Data presented thus suggest hepatocytes, besides mononuclear phagocytes, as possible source of IL-36γ during inflammatory liver damage. To further investigate the relevance of IL-36γ for liver pathology, expression of IL-1Rrp2, the decisive IL-36 receptor chain, was analyzed. Liver tissue IL-1Rrp2 mRNA was significantly upregulated 6 h after treatment of mice with APAP (264 ± 45% *versus* control mice (ctrl, see methods section) set as 100%, means ± SEM; n = 6, p = 0.0075, Student's t-test). At the 24 h time point after APAP administration, hepatic IL-1Rrp2 mRNA levels had decreased to levels not significantly different from those of ctrl mice (data not shown). Subsequent immunohistochemical analysis revealed that augmented IL-1Rrp2 mRNA levels detectable in the early phase of intoxication associated with enhanced protein expression at the 24 h time point after APAP administration. IL-1Rrp2 protein was barely detectable in healthy murine liver. Notably, IL-1Rrp2 protein was located at the edges of centrilobular necrosis, a typical hallmark of APAP intoxication (Fig. 1e). Altogether, data indicate IL-36 responsiveness of the inflamed murine liver.

### Expression of CCL20 in APAP-induced liver injury

The C-C chemokine CCL20, also coined *liver and activation regulated chemokine*, is among genes efficiently upregulated by IL-36γ in human primary keratinocytes^26^,^28^,^40^ and bronchial epithelial cells^29^. Herein, we extend those observations to human, IL-1Rrp2 expressing (data not shown), Huh7 hepatocellular carcinoma cells (4.0-fold induction of CCL20 mRNA, 4 h incubation with IL-36γ at 100 ng/ml, n = 3, p = 0.0348, Student's t-test) and human monocyte-derived macrophages (463-fold induction of CCL20 mRNA, 4 h incubation with IL-36γ at 100 ng/ml, n = 5, p = 0.0012, Student's t-test), respectively.

**Figure 2:**
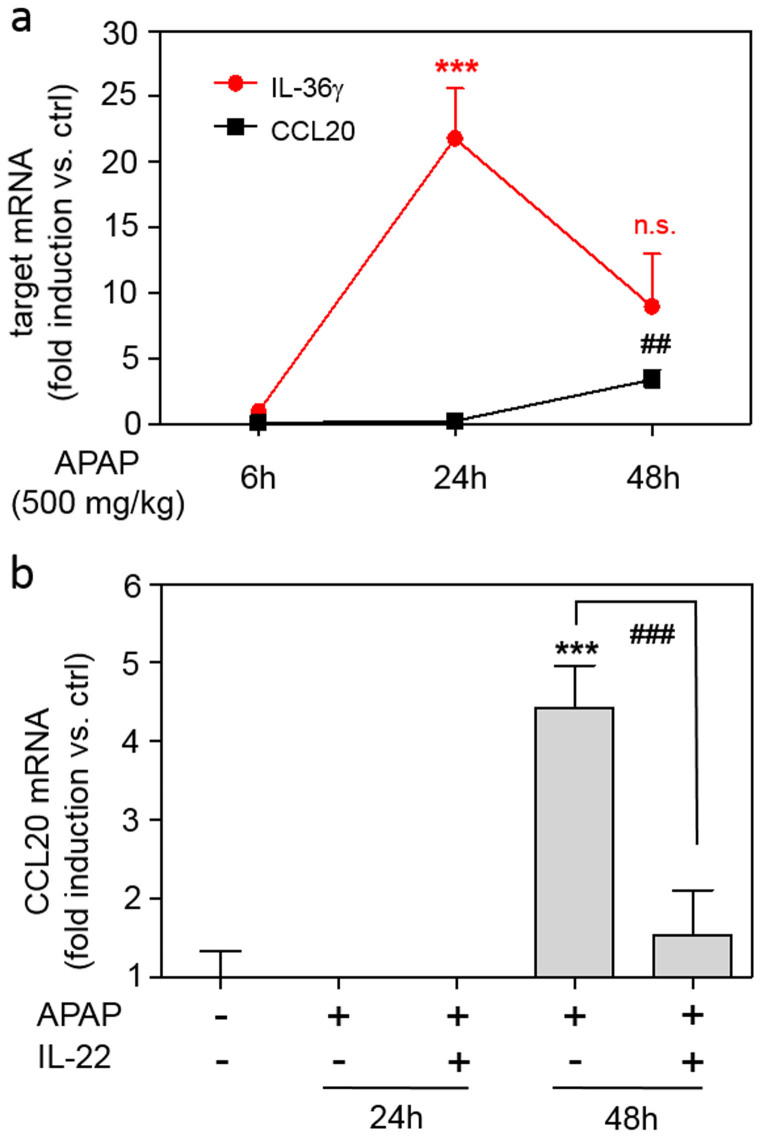
CCL20 expression in murine APAP-induced liver injury. (a) Mice received PBS (n = 6) or APAP (6 h, n = 6; 24 h, n = 9; 48 h, n = 11) for indicated time points. Hepatic CCL20 and IL-36γ mRNA was determined by realtime PCR. Target mRNA was normalized to that of GAPDH (means ± SEM *versus* ctrl of target mRNA, ***p < 0.001 and ##*p* < 0.01 *versus* ctrl at the respective time point). (b) Mice received PBS (n = 6) or APAP (24 h (n = 9) or 48 h (n = 6)) or APAP/IL-22 (24 h (n = 9) or 48 h (n = 6)). Hepatic CCL20 mRNA was determined by realtime PCR. Target mRNA was normalized to that of GAPDH (means ± SEM *versus* ctrl; ***p < 0.001 *versus* ctrl, ###*p* < 0.001). (ab) Statistical analysis, one-way analysis of variance with post hoc Bonferroni correction).

Since CCL20 has been related to hepatic necroinflammation^41^, we set out to investigate this chemokine in the context of APAP-induced liver injury. Compared to IL-36γ, CCL20 expression was induced with a delayed kinetic showing up well after 24 h of exposure to APAP (Fig. 2a). Amelioration of disease by application of IL-22 was, in similarity to observations on IL-36γ, associated with significant reduction of hepatic CCL20 expression (Fig. 2b).

### Application of IL-36Ra decreases CCL20 expression but increases parameters of tissue damage in late murine APAP-induced liver injury

**Figure 3:**
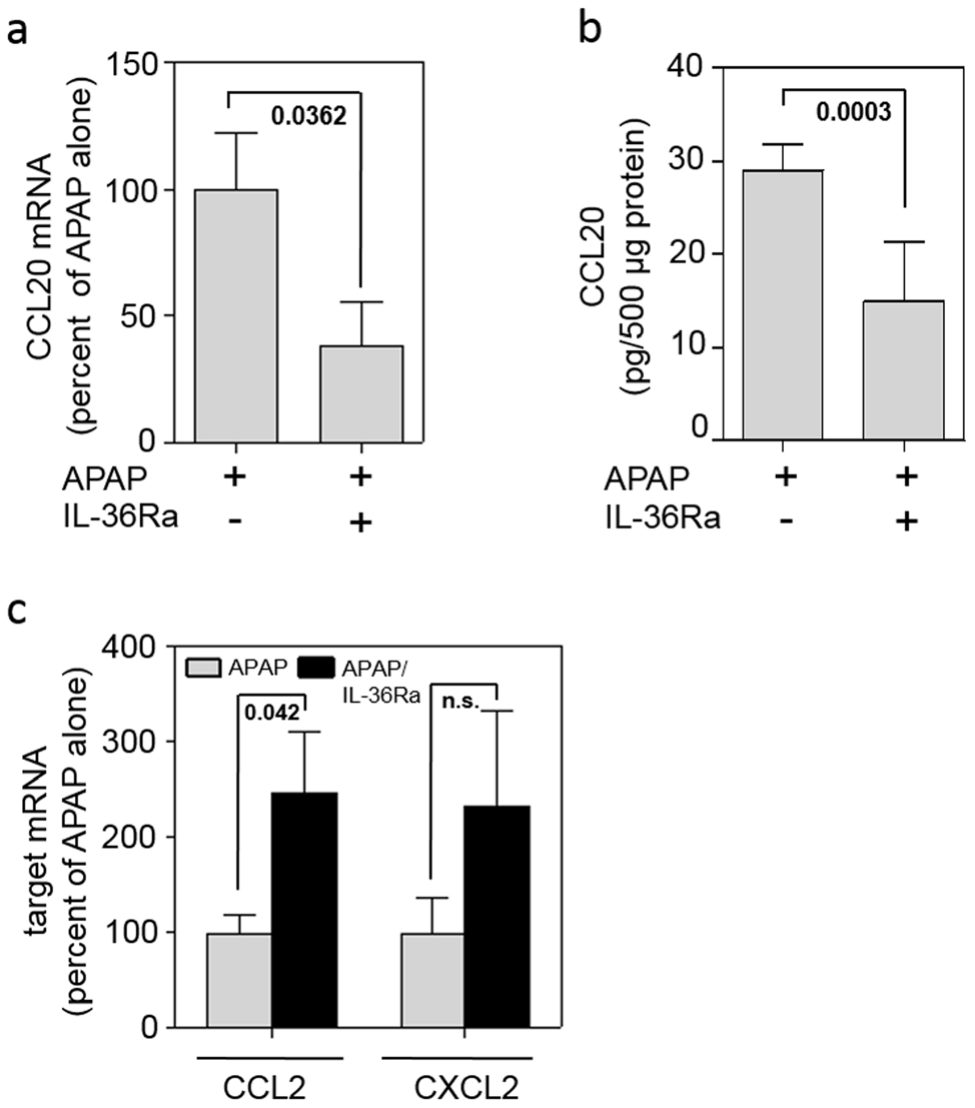
Application of IL-36Ra modulates CCL20 expression in late murine APAP-induced liver injury. (abc) Mice received APAP (n = 11) or APAP/IL-36Ra (n = 12) and were maintained for 48 h. Thereafter, hepatic CCL20 mRNA (a) and protein (b) was determined by realtime PCR and ELISA, respectively. (a) Target mRNA was normalized to that of GAPDH and is shown as percent of APAP alone (means ± SEM). (b) Liver tissue CCL20 content was determined by ELISA, is depicted as pg/500 μg total protein, and shown as means ± SEM. (c) Hepatic CCL2 and CXCL2 mRNA was determined by realtime PCR. Target mRNA was normalized to that of GAPDH and is shown as percent of APAP alone set as 100% (means ± SEM). Statistical analysis, Student's t-test; n.s., not significant.

To assess the function of IL-36γ in liver pathology, mice were co-treated with recombinant murine IL-36Ra along with APAP administration. After 48 h, hepatic chemokine expression and liver damage as detected by histology and serum alanine aminotransferase (ALT) activity was evaluated. In accord with data demonstrating induction of CCL20 by recombinant IL-36γ in cell culture, significant reduction of hepatic CCL20 expression became evident in mice that underwent APAP toxicity upon treatment with IL-36Ra. Reduction of CCL20 by IL-36Ra was detected on mRNA (Fig. 3a) and protein level (Fig. 3b), respectively. Notably, this was not the result of a general anti-inflammatory effect of IL-36Ra, because hepatic expression of CCL2 and CXCL2, both markers of local hepatic inflammation, was either augmented or unaffected upon IL-36Ra (Fig. 3c).

**Figure 4:**
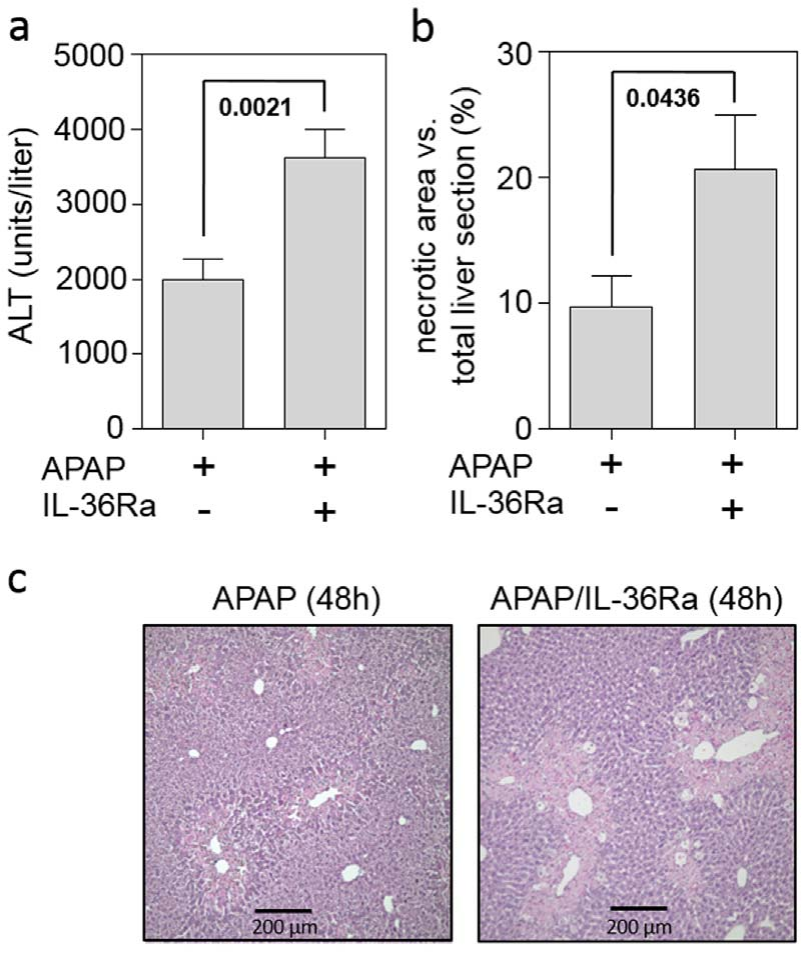
Application of IL-36Ra exacerbates tissue damage in late murine APAP-induced liver injury. (a) Mice received either APAP (n = 16) or APAP/IL-36Ra (n = 16) and were maintained for 48 h. Thereafter, serum ALT activity was determined and is depicted as units/liter (means ± SEM). (b) Mice received either APAP (n = 8) or APAP/IL-36Ra (n = 8). Statistical analysis of necrotic areas in H&E-stained liver sections after 48 h. (ab) Statistical analysis, Student's t-test. (c) Representative liver sections (H&E staining) 48 h after the onset of APAP intoxication.

To determine the functional relevance of IL-36γ blockage during APAP-induced liver injury, serum ALT was analyzed. While not significantly affecting serum ALT near the peak of hepatic injury at 24 h after APAP administration [8683 ± 1123 units/liter (n = 10) *versus* 10221 ± 1744 (n = 9) for APAP versus APAP/IL-36Ra, respectively], IL-36Ra application significantly upregulated this parameter of liver injury in the late phase of intoxication at the 48 h time point (Fig. 4a). Accordingly, computer-based analysis of histological liver sections demonstrated a significant 2-fold increase of APAP-induced necrosis upon IL-36Ra treatment (Fig. 4b). Fig. 4c displays representative 48 h time point liver sections derived from mice undergoing either APAP (left panel) or APAP/IL-36Ra treatment (right panel). Compensatory proliferation in late APAP toxicity was assessed by immunohistochemical analysis of Ki67, an established marker of hepatocyte proliferation. As compared to mice obtaining APAP alone, administration of IL-36Ra to APAP treated-mice was associated with a significant 21.5 ± 3.8% decrease of Ki67-positive hepatocytes (n = 8 for both groups, p = 0.0155, Students t-test) indicating some reduction of compensatory proliferation under the influence of IL-36Ra.

### IL-36γ expression in livers of endotoxemic mice

To extend current information on hepatic IL-36γ expression to inflammatory conditions different from APAP-induced liver injury, murine endotoxemia was analyzed. As shown in table 1, endotoxemia mediated, along with upregulation of prototypic markers of inflammation such as TNFα, IL-1β, and IL-6, strong induction of hepatic IL-36γ and CCL20 mRNA expression.

## Discussion

Analysis of hierarchically organized cytokine networks is key to advance our understanding of diseases that associate with inflammatory processes. Here, we propose IL-36γ as novel player in late APAP-induced liver damage. Using murine endotoxemia, we confirm expression of IL-36γ by the diseased liver.

Expression of IL-36γ in murine liver was detectable at 24 h after APAP administration, proposing that this cytokine regulates a later phase of intoxication, which may coincide with tissue repair and regeneration. At that time point, IL-1Rrp2, the IL-36 receptor, was increased at the edges of centrilobular necrosis localizing IL-36γ action specifically to the site of extensive hepatic injury. Amelioration of APAP-induced toxicity by administration of IL-22 reduced hepatic IL-36γ expression. Altogether, these observations suggest that release of alarmins from necrotic liver parenchyma in the amplification/injury phase of intoxication^15^ mediates somewhat delayed expression of IL-36γ, possibly secondary to more proximal inflammatory cytokines. In fact, induction of IL-36γ was detected in cultured murine primary hepatocytes or human Huh7 hepatocellular carcinoma cells exposed to inflammatory IL-1/TNFα/IFNγ. Beyond parenchymal cells such as hepatocytes, we recently reported on IL-36γ expression by activated mononuclear phagocytes^26^. It should be noted that the role of Kupffer cells and other macrophage lineages in APAP toxicity is not discussed uniformly. Whereas mononuclear phagocytes may contribute to early liver injury during initial intoxication^42^, those cells evidently initiate and promote the regeneration phase of intoxication^15^.

On a functional level, we demonstrate that application of recombinant IL-36Ra significantly aggravates late phase liver damage, as detected by histological analysis of liver necrosis and serum ALT activity. Moreover, we observed that IL-36γ determines induction of CCL20 in this advanced phase of liver injury. This conclusion is based on impaired CCL20 mRNA and protein expression after IL-36Ra administration. IL-36Ra is a genuine receptor antagonist that blocks activity of IL-36 by binding to IL-1Rrp2^24^. However, activation of the orphan receptor SIGIRR was recently put forward as additional mode of IL-36Ra action. SIGIRR curbs signaling by TLR ligands or IL-1 cytokines thus inhibiting key inflammatory pathways, among others NF-κB- and c-jun terminal kinase (JNK)-dependent signalling^43^. A connection between SIGIRR and IL-36Ra was proposed in light of early data indicating that upregulation of anti-inflammatory IL-4 by IL-36Ra in the brain is abrogated in SIGIRR knockout mice^44^. However, biochemical evidence supporting direct binding of IL-36Ra to SIGIRR has not been presented. Several points yet argue against SIGIRR as regulatory mechanism in the context of data presented herein. First, activation of anti-inflammatory SIGIRR would likely ameliorate APAP-induced liver injury. This assumption also connects to the pathogenic role of JNK in APAP intoxication^45^ and the capability of IL-4 to ameliorate disease in this setting^46^. In fact, we actually observed significantly augmented late histological necrosis and serum ALT activity, a systemic parameter reflecting liver damage, in IL-36Ra treated mice. Accordingly, IL-36Ra administration likewise associated with enhanced hepatic expression of CCL2, a notorious surrogate of necroinflammation. Altogether, data indicate that specific downregulation of CCL20 by IL-36Ra is unlikely related to SIGIRR but mediated by blockage of IL-36γ on the level of IL-1Rrp2. In accord with the requirement of IL-36γ biological activity for efficient CCL20 induction *in vivo*, we observed upregulation of this chemokine by IL-36γ in cultured Huh7 hepatocellular carcinoma cells and primary macrophages.

Regulation of CCL20 by IL-36γ may impact on the course of APAP-induced disease. CCL20 is a C-C chemokine targeting CCR6 as sole receptor. The complex biology of CCL20 is largely based on functional CCR6 expression on T cell subsets with partly opposing functions in pathophysiology, namely regulatory T cells (Tregs), Th17 cells^47^, and γδ T cells^48^. γδT cells have been described to aggravate APAP-induced liver injury by producing IL-17^8^. A similar mode of pathogenic action supposedly applies to Th17 cells that likewise accumulate in the liver upon APAP intoxication^49^. However, the liver-homing capacity of CCL20 may also mediate tissue protection, repair, and regeneration. Notably, a recent report demonstrates that the aforementioned CCL20-γδT cell-IL-17-IL-22-axis likewise promotes liver regeneration after experimental murine hepatectomy^50^. These observations suggest that, in addition to differences in disease models, timing of γδT cells action may be pivotal for their function on the course of liver injury. Although the function of Tregs has, to the best of our knowledge, not been reported so far for APAP intoxication, their protective potential in acute liver injury is evident^51^. In fact, IL-10, a major Treg product, is protective in APAP-induced liver damage^52^. CCL20 may also directly stimulate hepatic stellate cells (HSC), for example by activating pro-proliferative mitogen-activated protein kinases^53^. Notably, depletion of HSC worsens experimental APAP intoxication^54^. The relevance of CCL20 acting on HSC for repair in APAP-induced liver injury has not been investigated. However, as HSC are crucial for liver regeneration^55^, their activation by CCL20 might be of significance.

Finally, aggravation of APAP-induced liver damage by IL-36Ra is reminiscent of data on amelioration of toxicity detected in IL-1Ra deficient mice^21^. In fact, it has been shown that IL-1 produced by necrotic liver tissue is able to support regeneration by compensatory hepatocyte proliferation *via* activation of the IL-6/STAT3 axis^56^. IL-6, along with IL-22, is key to STAT3 activation in diseased liver tissue^57^. Notably, IL-36γ has been shown to efficiently induce IL-6 in mononuclear phagocytes^27^,^40^ and endogenous IL-6 displays protective/pro-regenerative action in APAP-induced liver injury^58^,^59^. Herein, application of IL-36Ra reduced hepatocyte Ki67 expression in the late regeneration phase of APAP-induced liver injury by 21%. Albeit this effect appears modest, it indicates that impaired compensatory proliferation may contribute to aggravation of toxicity upon IL-36 blockage. Observations suggest that, when biologically active, IL-36γ likely contributes to a mediator milieu favoring tissue repair/regeneration in the later phase of APAP intoxication.

Altogether, the unexpected observation that blockage of IL-36γ by IL-36Ra exacerbates APAP-induced liver injury may reflect the counterintuitive but inherent potential of some inflammatory cytokines to initiate regeneration when adequately expressed at the right time and place.

## Methods

### Reagents

APAP and lipopolysaccharide (LPS; O55:B5) were from Sigma-Aldrich, Taufkirchen, Germany. Murine IL-22 was from Immunotools, Friesoythe, Germany. Mature human IL-36γ (aa18-aa169) and murine IL-36Ra were from R&D Systems, Wiesbaden, Germany. IL-1β (human), TNFα (human, mouse), IFNγ (human, mouse), and human macrophage colony-stimulating factor (M-CSF) were from Peprotech Inc., Frankfurt, Germany.

### Murine APAP-induced liver damage and experimental endotoxemia

Experiments using male C57Bl/6N mice (Charles River Laboratories, Sulzfeld, Germany; 8–10 weeks old) were approved by the Regierungspräsidium Darmstadt and were in accordance with NIH guidelines. Before being sacrificed, mice underwent short isoflurane (Abbott, Wiesbaden, Germany) anesthesia. Blood was drawn from the retro orbital venous plexus. Serum ALT activity was quantified according to manufacturer's instructions (Reflotron, Roche Diagnostics, Mannheim, Germany). For RNA analysis, liver tissue was snap frozen in liquid nitrogen and stored at −80°C. For histological analysis, liver tissue was perfused with PBS via the portal vein followed by overnight incubation in 4.5% buffered formalin. Paraffin-embedded liver sections (4 μm) were stained with H&E. Histopathologic liver injury was quantified in blinded manner by ImageJ 1.46r (NIH, Bethesda, MD) software. Murine APAP-induced liver damage was induced as previously described^34^. Briefly, fasted male mice obtained (as indicated) i.v. injections of PBS or IL-22 (3.5 μg/mouse) or IL-36Ra (6 μg/mouse). Immediately thereafter, i.p. injection of either warm NaCl (0.9%, B.Braun, Melsungen, Germany) or APAP (500 mg/kg dissolved in warm 0.9% NaCl) was performed. Mice that obtained PBS followed by NaCl are depicted as control mice (ctrl) throughout the manuscript. Mice had free access to food and water thereafter. After indicated time points, mice were sacrificed. We experienced a relatively low 48 h-mortality rate in this model of murine APAP (500 mg/kg)-induced liver injury (2/24 for APAP and 1/17 for APAP/IL-36Ra, respectively) which, however, agrees with a recent report using a very similar experimental protocol^17^. Notably, at the higher 750 mg/kg APAP dosage we observed an approximately 50% mortality rate within a 24 h observation period (4/9)^34^.

For induction of endotoxemia, unfasted male mice were injected intraperitoneally with either PBS or LPS (5 mg/kg dissolved in PBS). After three hours, mice were sacrificed and hepatic cytokine expression was analyzed.

### Immunohistochemical detection of IL-1Rrp2 and Ki67 in liver tissue

For detection of hepatic IL-1Rrp2- and Ki67 positive cells, paraffin-embedded liver sections (4 μm) were used. Sections were deparaffinized and unmasked by heat treatment (Dako Target Retrieval Solution, Glostrup, Denmark). Thereafter, sections were incubated with goat anti-murine IL-1Rrp2 antibody (R&D Systems, Wiesbaden, Germany) or rabbit anti-murine Ki67 antibody (Epitomics, Burlingame, CA) overnight at 4°C. For detection, rabbit anti-goat universal immuno-peroxidase polymer (IL-1Rrp2) or goat anti-rabbit universal immune-peroxidase polymer (Ki-67; both Nichirei Biosciences, Tokyo, Japan) and DAB Substrate Kit for Peroxidase (Vector Laboratories, Burlingame, CA, USA) were used. Sections were counterstained using hematoxylin.

### Isolation of murine hepatocytes

For isolation of murine hepatocytes C57Bl/6N mice were anesthetized with ketamine (100 mg/kg, i.p.) and xylazine (10 mg/kg, i.p.). After disinfection and laparotomy, a 22 G cannula was placed in the inferior vena cava. After dissection of the portal vein, retrograde liver perfusion was performed with 37°C warm HBSS without Ca^2+^ and Mg^2+^ (supplemented with 15 mM HEPES, 2.5 mM EGTA, 1 g/l glucose, 100 U/ml penicillin, and 100 μg/ml streptomycin) using a roller pump (10 ml/min) for 10 min. Next, the liver was perfused with HBSS with Ca^2+^ und Mg^2+^ (supplemented with 15 mM HEPES, 5 mM CaCl_2_, 0.13 mg/ml collagenase IV (Sigma-Aldrich), 100 U/ml penicillin, and 100 μg/ml streptomycin) for additional 10 min. The liver was carefully removed from the abdominal cavity, placed in a Petri dish on ice and opened with a forceps. Liver cells were resuspended in Williams Medium E (supplemented with 10% FCS, 2 mM L-Alanyl-L-Glutamin (Biochrom, Berlin, Germany), 100 U/ml penicillin, and 100 μg/ml streptomycin) and put over a 100 μm cell strainer (Becton Dickinson, Franklin Lakes, NJ, USA). After two rounds of centrifugation (5 min at 50 g and 4°C) and resuspension, cell viability was determined by trypan blue dye exclusion, and cells were seeded in serum-free medium on collagen G-coated plates (Biochrom). Adherent hepatocytes were washed after 2 h with PBS and fresh Williams Medium E (supplemented with 10% FCS, 2 mM L-Alanyl-L-Glutamin, 100 U/ml penicillin, and 100 μg/ml streptomycin) was added. Stimulation with recombinant cytokines was performed 16 h thereafter.

### Generation of human monocyte-derived macrophages

For isolation of peripheral blood mononuclear cells (PBMC), written informed consent was obtained from healthy donors, and blood was taken. All experimental protocols were approved by the ‘Ethik Kommission' of the University Hospital Goethe-University Frankfurt. The methods were carried out in accordance with the approved guidelines. Healthy donors had abstained from taking drugs/medication for 2 weeks before the study. PBMC were isolated from peripheral blood using Histopaque-1077 (Sigma-Aldrich) according to the manufacturer's instructions. The CD14^+^ cell population of PBMC was isolated by using CD14 antibody-conjugated magnetic microbeads (Miltenyi, Bergisch-Gladbach, Germany). After isolation, CD14^+^ cell purity was determined by FACS analysis (FACS Canto, BD Biosciences, Heidelberg, Germany) using a mouse monoclonal anti-human CD14-phycoerythrin antibody (eBioscience, Frankfurt, Germany). Enrichment of CD14^+^ cells was >94.9%. Cells were resuspended in IMDM (supplemented with 2% heat-inactivated human serum (Life Technologies, Darmstadt, Germany), 100 U/ml penicillin, 100 μg/ml streptomycin, 1× nonessential amino acids, and 50 μM 2-mercaptoethanol) and seeded at a density of 8× 10^5^ cells/0.75 ml on 12-well polystyrene plates (Greiner, Frickenhausen, Germany). For generation of monocyte-derived macrophages, CD14^+^ cells were cultured in the presence of M-CSF (100 ng/ml) for 7 days. 75% of spent medium was exchanged for fresh medium and fresh cytokines on days 3 and 5. On day 7 macrophages were stimulated in the aforementioned medium as indicated.

### Cultivation of Huh7 cells

The human hepatocellular carcinoma cell line Huh7 (kindly provided by Dr. Kai Breuhahn, Institute of Pathology, University of Heidelberg, Germany) was maintained in DMEM (with 100 U/ml penicillin, 100 μg/ml streptomycin, and 10% FCS)^60^. For experiments, cells were seeded on 6-well polystyrene plates in the aforementioned culture medium. All incubations were performed at 37°C and 5% CO_2_.

### Detection of IL-36γ by immunoblot analysis

Cell lysates were obtained as previously described^26^. Briefly, for detection of proteins, cells were lysed in lysis buffer (150 mM NaCl, 1 mM CaCl_2_, 5 mM Tris-Cl (pH 7.4), 1% Triton X-100), supplemented with protease inhibitor cocktail (Roche Diagnostics, Mannheim, Germany) and DTT, Na_3_VO_4_, PMSF (each 1 mM), and NaF (20 mM). Antibodies: IL-36γ, goat polyclonal antibody (R&D Systems); β-Tubulin, mouse monoclonal antibody (Santa Cruz Biotechnology).

### Analysis of cytokine expression by realtime PCR

After RNA isolation (Tri-Reagent, Sigma-Aldrich), 1 μg of total RNA was transcribed using hexameric primers and Moloney virus RT (Life Technologies). Realtime PCR was performed on AbiPrism 7500 Fast Sequence Detector (Life Technologies): one initial step at 95°C for 5 min was followed by 40 cycles at 95°C for 2 sec and 60°C for 25 sec. Detection of the dequenched probe, calculation of threshold cycles (Ct values), and data analysis were performed by the Sequence Detector software. The following probes (Applied Biosystems) were used (all FAM): mIL-36α, Mm00457645_m1; mIL-36β, Mm01337546_g1; mIL-36γ, Mm00463327_m1; mIL-1Rrp2, Mm00519245_m1; mCCL20, Mm01268754_m1; mCCL2, Mm00441242_m1; mCXCL2, Mm00436450_m1; mTNFα, Mm00443285_m1; mIL-1β, Mm00434228_m1; mIL-6, Mm00446190_m1; hCCL20, Hs01011368_m1; hIL-36γ, Hs00219742_m1. mRNA expression was normalized to GAPDH (mouse: 4352339E; human: 4310884E; both VIC) and relative changes in the respective mRNA were quantified by the 2^−ddCt^ method.

### Analysis of cytokine release by enzyme-linked immunosorbent assay (ELISA)

Murine CCL20 (hepatic protein lysate) was determined by ELISA according to the manufacturer's instructions (R&D Systems). For detection of CCL20 in hepatic tissue, liver specimens were homogenized in lysis buffer (150 mM NaCl, 1 mM CaCl_2_, 5 mM Tris-Cl (pH 7.4), 1% Triton X-100), supplemented with protease inhibitor cocktail (Roche Diagnostics, Mannheim, Germany) and DTT, Na_3_VO_4_, PMSF (each 1 mM), and NaF (20 mM). To control for background effects, ELISA standard curve and blank were prepared in corresponding dilutions of lysis buffer.

### Statistical analysis

Data were first evaluated with the Kolmogorov-Smirnov test for parametric distribution. For two groups, raw data were analyzed by an unpaired two-tailed Student's t-test. For comparison of three or more groups, raw data were analyzed by one-way analysis of variance with post hoc Bonferroni correction. Data are shown as means ± standard error of the mean (SEM, animal data or cell culture using primary cells) or as means ± standard deviation (SD, cell culture data using cell lines) and are presented as fold induction, pg/500 μg total protein, % (APAP vs. APAP/IL-36Ra; necrotic area vs. total liver section), units/liter, or as raw data (2^−ddCt^ × 10^7^). Differences were considered statistically significant if the p value was <0.05 (GraphPad Prism, La Jolla, CA, USA). For analysis by unpaired Student's t-test specific p values are indicated whereas in case of analysis by one-way analysis of variance upper limits of p values are depicted.

## Author Contributions

P.S., M.B., L.H. and T.P. performed experiments. P.S., M.B., A.P., B.Z., J.P. and H.M. analyzed the data and contributed in manuscript editing and relevant citations. P.S. and M.B. prepared the figures. P.S. and H.M. designed the study. H.M. wrote the paper. All authors discussed the results and reviewed the manuscript.

## Figures and Tables

**Table 1:** Fold induction of hepatic cytokine mRNA expression after 3h of endotoxemia *versus* untreated ctrl animals

**IL-36γ**	41.5 ± 7.0 p = 0.0002
**CCL20**	51.6 ± 16.6 p = 0.0123
**TNFα**	28.2 ± 7.9 p = 0.0065
**IL-1β**	30.3 ± 9.7 p = 0.0127
**IL-6**	67.9 ± 19.4 p = 0.0062

Data are shown as means ± SEM.

p-values compared to ctrl animals.
